# Emotional words facilitate lexical but not early visual processing

**DOI:** 10.1186/s12868-015-0225-8

**Published:** 2015-12-12

**Authors:** Sophie M. Trauer, Sonja A. Kotz, Matthias M. Müller

**Affiliations:** Lehrstuhl für Allgemeine Psychologie, Institut für Psychologie, Universität Leipzig, Neumarkt 9-19, 04109 Leipzig, Germany; Department of Neuropsychology and Psychopharmacology, Faculty of Psychology and Neuroscience, Maastricht University, Maastricht, The Netherlands; Department of Neuropsychology, Max Planck Institute for Human Cognitive and Brain Sciences, Leipzig, Germany

**Keywords:** Emotion, Language, Visual attention, SSVEP, ERP, LDT

## Abstract

**Background:**

Emotional scenes and faces have shown to capture and bind visual resources at early sensory processing stages, i.e. in early visual cortex. However, emotional words have led to mixed results. In the current study ERPs were assessed simultaneously with steady-state visual evoked potentials (SSVEPs) to measure attention effects on early visual activity in emotional word processing. Neutral and negative words were flickered at 12.14 Hz whilst participants performed a Lexical Decision Task.

**Results:**

Emotional word content did not modulate the 12.14 Hz SSVEP amplitude, neither did word lexicality. However, emotional words affected the ERP. Negative compared to neutral words as well as words compared to pseudowords lead to enhanced deflections in the P2 time range indicative of lexico-semantic access. The N400 was reduced for negative compared to neutral words and enhanced for pseudowords compared to words indicating facilitated semantic processing of emotional words. LPC amplitudes reflected word lexicality and thus the task-relevant response.

**Conclusion:**

In line with previous ERP and imaging evidence, the present results indicate that written emotional words are facilitated in processing only subsequent to visual analysis.

## Background

Emotional signals are favored in perception as seen in behavioral performance and corresponding cortical activity [1, 2]. Emotional scenes and faces have been demonstrated to facilitate processing already in early visual cortex, e.g. [3–5]. However, in the case of symbolic stimuli such as written words (for reviews, see [6, 7]) it is still a matter of debate which processing stages are facilitated by emotional content: Are arousing signals favored in an ‘automatic’ way along all processing stages including sensory (re-) processing? Or does emotional meaning shape only more fine-grained, object-specific analysis steps, i.e. the lexico-semantic analysis of word forms? In order to address this question, we combined the analysis of event-related potentials (ERPs) in response to neutral and emotional words with a continuous measure of neural activity in early visual cortex, the steady-state visual evoked potential (SSVEP).

Previous studies on ERP differences between emotional and neutral words provide mixed results about the involvement of early visual areas in processing advantages for linguistic emotional material. Only few studies report emotion effects as early as the P1 [8–10]. ERP modulations by emotional words are often found only after 200 ms, e.g. [6, 7, 11–13], raising the question whether emotional word content facilitates early visual processing.

A robust finding with emotional words is enhanced neural activity in the P2 time range [6, 7] denoting a processing stage where complex visual information, i.e. word forms are matched with semantic knowledge [6, 14]. At this latency, many studies report an enhanced early posterior negativity in response to emotional stimuli, e.g. [15, 16]. Others report an enhanced frontal positivity in response to emotional words [17, 18]. In order to adress both lines of research, we chose an analysis approach comprising all four quadrants of electrode space. Emotional word content has also repeatedly been shown to affect later processing stages in the N400 and LPC range [6, 7]. These late components reflect more elaborate processing and effects on their amplitude and latency seem more dependent on paradigm, context, and task requirements [7, 19–21].

While the ERP provides detailed information of post-stimulus processing at different stages, the SSVEP is an ongoing oscillatory brain response to a flickering visual stimulus that is generated in early visual cortex [22]. Attention leads to its amplitude rise [23–25]; therefore SSVEPs provide a powerful tool to study the neural dynamics of attentional resource allocation in early visual cortex. Previous research demonstrated that emotional compared to neutral scenes and faces capture visual processing resources as reflected in the SSVEP amplitude [4, 23, 26, 27]. So far, only few studies used frequency tagging to study attentional resource allocation to written words as a function of affective valence. Koban et al. [28] flickered words at 7.5 Hz during free viewing and found a sustained decrease of the 7.5 Hz SSVEP amplitude elicited by positive compared to neutral and negative words when they analyzed the signal of the entire stimulation period of about 8 s. The authors interpreted this somewhat surprising result as evidence for increased internal processing that results in reduced activity early visual cortex. Contrary to this finding, Keil and coworkers [29] reported increased SSVEP amplitudes at 120 to 270 ms after word onset for emotional compared to neutral words. The authors used an attentional blink (AB) paradigm with rapid serial visual presentation (RSVP) at a rate of 8.6 Hz. Participants had to read and recognize words of a target color in an ongoing visual stream. However, the SSVEP emotion effect was limited to second targets (T2) in the ‘lag 2’ condition, i.e. when only one stimulus occurred between the first target (T1) and the emotional or neutral T2. For T2 stimuli presented at later ‘lags’ there was no statistically significant difference. The notion that greater attentional resources were allocated to emotional T2 words was supported by better word identification rates for emotional T2 stimuli. In contrast, in a recent study [21] we found no greater distraction effect from a visual foreground task for emotional compared to neutral word distractors. Participants were instructed to attend to a cloud of flickering squares (eliciting the SSVEP) and to detect short coherent motion events of a subset of the squares. Emotional or neutral words were presented at unpredictable latency in the background of the task display. If emotional words attracted attentional resources as indicated by Keil [29] and as demonstrated for emotional images in similar experiments, e.g. [26, 27], we would have expected a greater reduction of SSVEP amplitudes for emotional compared to neutral distractors. However, neither SSVEP amplitudes nor task performance differed between neutral and emotional background words. Taken together, the few studies that looked into neural dynamics of emotional word processing using the SSVEP have provided inconsistent, if not surprising results. One reason may be the different experimental designs that were either linked to no task [28], an AB task [29], or a foreground task with words as distractors [21]. It may well be the case that task instructions influence at which level of word processing attentional facilitation of emotional words will occur. Only the study by Keil and colleagues [29] required participants to read the words in order to give a correct response. Accordingly, task-relevance, i.e. voluntary or top-down driven attention towards word stimuli, may be a prerequisite for the neural facilitation in early visual cortex. Emotional compared to neutral words may then deploy additional attentional resources resulting in increased SSVEP amplitudes.

In the present study we set out to investigate at which stages of word processing emotional words facilitate neural responses. To ensure that participants paid attention to the stimuli, we used a Lexical Decision Task (LDT) and assessed ERPs and SSVEP amplitudes simultaneously in order to disentangle emotion effects during early visual and later lexico-semantic processing. To separate ERP and SSVEP amplitudes in the frequency range, we flickered the stimuli at a frequency above 10 Hz and introduced a baseline in which flickering letter strings were presented that switched either to an emotional or neutral word or to a pseudoword. This baseline provides an estimate of the SSVEP amplitude without lexical processing on the one hand, and avoids that the stimulus onset produces an onset ERP contaminating the SSVEP response [28]. If emotional compared to neutral words result in neural facilitation of early visual cortex, we expect greater SSVEP amplitudes to emotional words. If, however, cortical facilitation is restricted to lexico-semantic and subsequent processing stages, we expected modulations of respective ERP components from 200 ms post-stimulus onwards [6, 7] without any effect upon SSVEP amplitudes. Furthermore, a comparison between words and pseudowords allowed relating possible emotion effects to lexical processing.

## Methods

Twenty volunteers (9 female, mean age 25.8 years, SD 4.4) participated in the experiment and received course credit or monetary compensation (6 € per hour). All had normal or corrected-to-normal vision, were right-handed, native speakers of German, and reported no difficulties of reading or spelling. Prior to the recordings, participants received information about the study goals and gave written informed consent. The study conformed to the Code of Ethics of the World Medical Association and the standards of the local ethics committee of the University of Leipzig.

### Stimuli

Word lists were chosen from the Leipzig affective norms for German [30]. 60 neutral, e.g. *Beruf* (profession), *Beweis* (evidence), *Phase* (phase), and 60 negative words, e.g. *Armut* (poverty), *Tumor* (tumor), *Folter* (torture), were selected that differed significantly in valence (t_118_ = 36.2, p < 0.001) and emotional arousal (t_118_ = −32.3, p < 0.001). Rated concreteness, letter and syllable length, and word frequency were matched between stimulus sets (all p > 0.2; http://wortschatz.uni-leipzig.de). For the LDT 120 pseudowords of matching letter and syllable length (e.g. *Nurle*, *Pusk*, *Tibang*) were created that had no systematic resemblance to meaningful words but followed the orthographic rules of the German language.

**Fig. 1:**
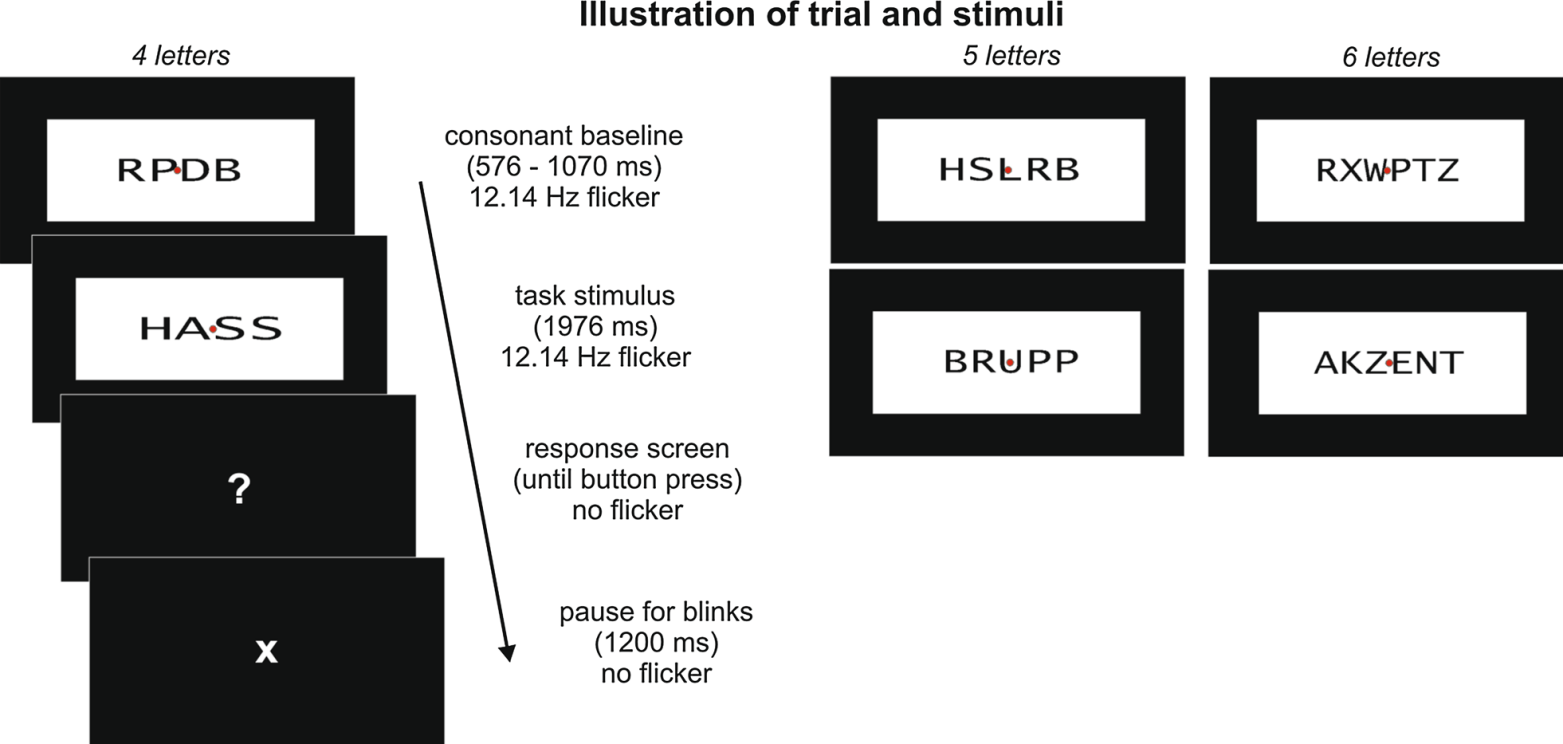
Stimuli and schematic presentation during the experiment. All word and pseudoword stimuli and corresponding consonant baseline stimuli had 4–6 letters and comprised a constant number of pixels in order to keep stimulus luminance constant between conditions (HASS: hate, BRUPP: pseudoword, AKZENT: accent)

All stimuli were paired with consonant strings serving as a flickering baseline to establish the SSVEP response prior to word onset. Stimuli were presented in black font within a white rectangle of a constant size of 15 by 6.5° of visual angle. The word form itself spanned approximately 11 × 4°. In order to control physical factors influencing SSVEP amplitudes, the luminance of stimuli was held constant, i.e. all letter strings were stretched horizontally to comprise a constant number of pixels (see Fig. 1 for an illustration of stimuli). A red fixation dot (0.3°) was presented in the centre of the screen throughout each trial. Stimuli were presented on a 19-inch CRT monitor at a viewing distance of 80 cm. The refresher rate of the monitor was set at 85 Hz.The flicker frequency of 12.14 Hz was realized by presenting the rectangles containing the letter strings with 3 frames on and 4 frames off the screen.

### Task and procedure

Participants were seated in a sound-attenuated chamber, the electrodes were applied, and the LDT was explained. Participants were instructed to avoid eye movements and button presses during the presentation and that the response (left or right Shift key counterbalanced across participants) was required and recorded only after each trial when a ‘?’ appeared on the screen.

All words were presented twice throughout the experiment resulting in a total of 480 experimental trials for further analyses. Words were presented in random order with the restriction that the same baseline duration or word type occurred in no more than three consecutive trials. All trials started with the presentation of a consonant baseline of randomized duration (576–1070 ms). The baseline was followed by a word or pseudoword for 1976 ms. Then the ‘?’ appeared on the screen and responses were registered between 200 and 1000 ms afterwards. Subsequently an ‘X’ indicated a pause to blink for 1200 ms before the next trial started. To prevent temporal expectation effects, 80 catch trials were presented with shorter baseline durations (247 or 412 ms). Stimuli for these catch trials were words from the same database (10 neutral, 10 negative, and 20 additional pseudowords), matched in length, but less distinctive in their valence and/or arousal ratings. These trials were not included in analyses. Before recording, the task was trained in two blocks of 30 trials with additional word and pseudoword stimuli. The recording session had 8 blocks of 70 trials each.

### EEG recording and processing

The electroencephalogram (EEG) was recorded from 64 Ag/AgCl scalp electrodes (see Fig. 3a for the electrode layout) at a sampling rate of 512 Hz using an ActiveTwo amplifier system (BioSemi, Amsterdam). Four additional electrodes recorded the horizontal and vertical electrooculogram. EEG data were processed using the ERPLAB plugin (http://erpinfo.org/erplab) running on MATLAB. Data epochs were extracted from −550 to 2000 ms around word onset. From each epoch the mean amplitude was subtracted relative to a zero mean baseline. Trials with eye movements or blinks were discarded. Two participants were excluded from further analyses because more than 30 % of trials were removed. For the remaining 18 datasets on average 16.2 (SD 5.6) percent of trials were excluded due to blinks, eye movements, or muscle activity. Artifacts such as noisy electrodes were corrected using a combination of channel approximation and epoch exclusion based on statistical parameters of the data with the ‘statistical control of artifacts in dense array EEG/MEG studies’ [31]. In the remaining trials on average 3.9 (SD 0.7) of 64 channels were interpolated. Data were then re-referenced to average reference. For each condition and participant the amplitudes of all trials were averaged for further analyses.

**Fig. 2:**
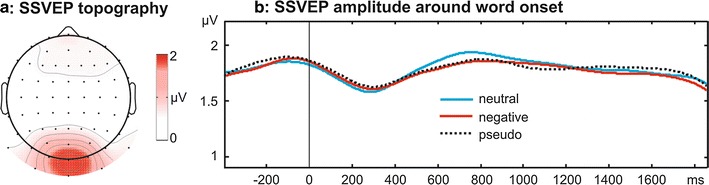
12.14 Hz steady-state visual evoked potential (SSVEP) elicited by flickering word stimuli. **a** Topography of the SSVEP amplitude from −450 to 1850 ms around word onset averaged across all conditions. Highest amplitudes and thus the individual best electrodes selected for analyses were centered around Oz. **b** SSVEP amplitude time courses around word *onset*. Conditions (neutral vs negative words, all words vs pseudowords) did not differ significantly at any sampling point

#### SSVEP analyses

In a first step the average amplitude of the 12.14 Hz signal from −400 to 1850 ms was calculated at each electrode with a Fast Fourier Transformation (FFT, implemented in Matlab). To improve the signal-to-noise-ratio we selected greatest SSVEP amplitudes across all conditions and the whole data epoch by picking the three individual best electrodes for each participant and averaged across these three electrodes for further analysis. All individual best electrodes were located close to Oz, reflecting the grand mean topography of SSVEP amplitudes (Fig. 2a). To extract the SSVEP amplitude time courses we applied a wavelet filter [32] centred at 12.14 Hz with a frequency resolution of ±1.47 Hz full-width at half-maximum resulting in a temporal resolution of ±150 ms. These time courses were tested by two running repeated measures ANOVAs (*Emotion*: neutral/negative, or *Lexicality*: words/pseudowords) at each sampling point from −400 to 1850 ms. Furthermore, we calculated an FFT for a time window between 0 and 1500 ms after word onset with the same electrodes as for the time course analyses. The resulting 12.14 Hz amplitudes were subject to two repeated measures ANOVAs comprising the factor *Emotion* (neutral, negative) or *Lexicality* (words, pseudowords), respectively.

#### ERP analyses

**Fig. 3:**
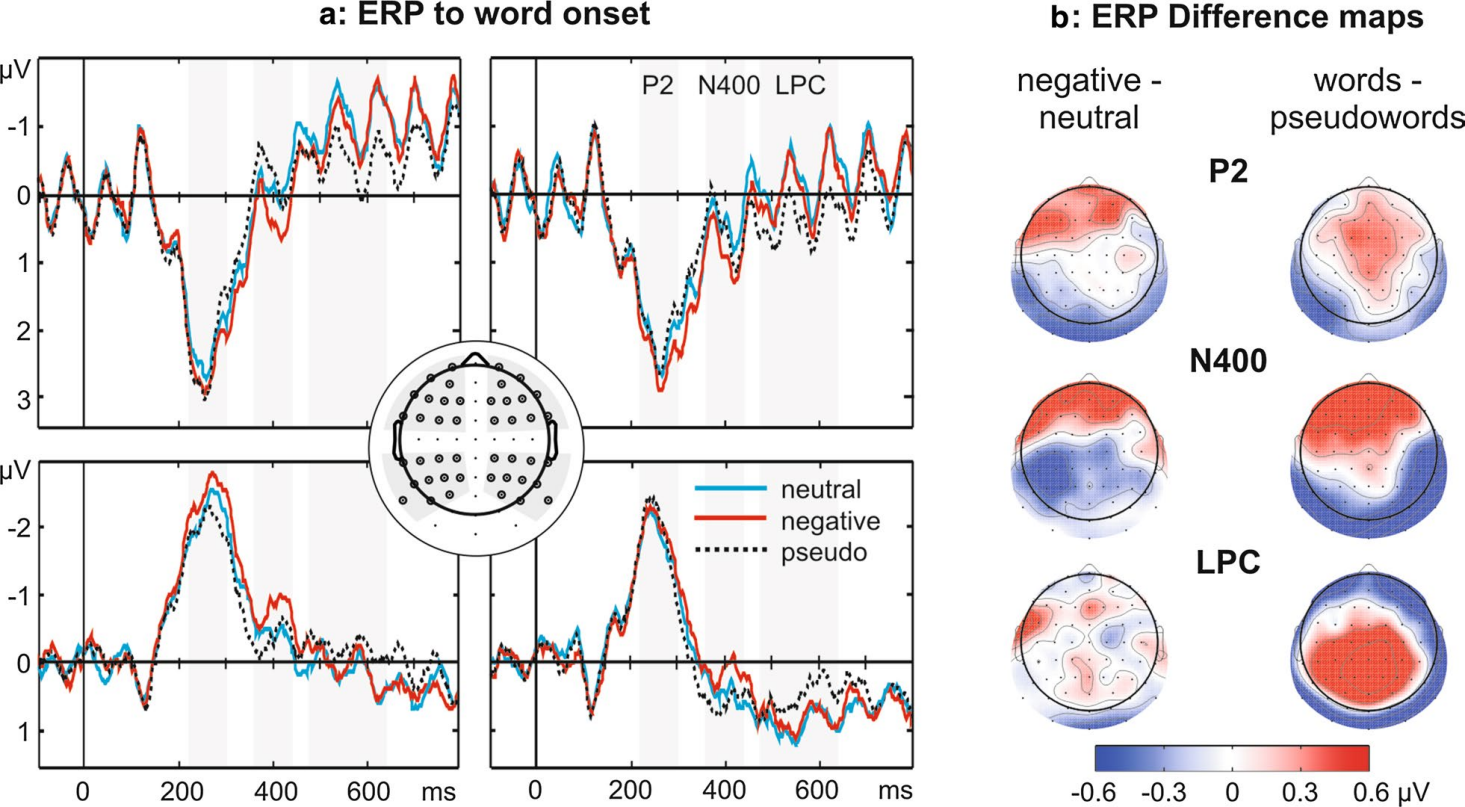
Event-related potential (ERP) to word *onset*. **a** ERPs at the four electrode clusters marked in the *middle panel*. *Gray bars* indicate analyzed time windows. Note that the visible SSVEP oscillation was not filtered out but its influence was minimized by analyzing time windows that were multiples of the 12.14 Hz SSVEP wavelength. Seemingly comparable SSVEP amplitudes at the four quadrants are a result of posterior clusters sparing the SSVEP maximum around Oz (Fig. 2a). **b** *Difference maps*: emotional word content (*left*) affected the ERP amplitude only during the P2 and N400 time windows, word lexicality (*right*) modulated all three components

The ERP to word onset was analyzed in the same dataset as the SSVEP amplitudes. ERP components were defined by inspection of the Global Field Power (GFP, implemented as standard deviation of amplitudes across all electrodes at a given sampling point). ERP amplitudes around GFP maxima were averaged within time windows that were multiples of the SSVEP frequency cycle (82.4 ms) thus minimizing an influence of the SSVEP oscillation on the ERP (see Fig. 3a and below for respective window latencies). Amplitudes were averaged across the sensors of four electrode clusters (Fig. 3a, middle panel) and then entered into repeated measures ANOVAs comprising three factors (*Region*: anterior, posterior; *Laterality*: left, right; *Emotion*: neutral, negative; or *Lexicality*: words, pseudowords). Results were Greenhouse-Geisser-corrected for sphericity. Significant effects were followed-up by two separate two-factorial ANOVAs within *Region*s (anterior or posterior sites: *Laterality* by *Emotion/Lexicality*) and paired-sample t-tests within electrode clusters.

## Results

### Behavioral Data

Hit rates in the LDT (mean 98.5 % ± SD 1.3 %) did not differ significantly between neutral (98.7 ± 1.5) and negative words (99.0 ± 1.2, t_16_ = −1.3, p > 0.2). Hit rates for words (98.8 ± 1.3) were slightly higher than for pseudowords (98.1 ± 1.7; t_16_ = 1.9, p = 0.07). Reaction times (2317.9 ms ± 86.4), obscured by the response delay, did not differ significantly between conditions (all |t_16_| < 1.2, p > 0.2).

### Steady state visual evoked potentials

Figure 2a shows the topographical distribution of SSVEP amplitudes across the entire trial and all experimental conditions and Fig. 2b depicts the SSVEP time course for the three stimulus categories. SSVEP amplitude was unaffected by emotional word content (all |F_17_| < 2.8, all p > 0.1) or *Lexicality* (all |F_17_| < 3.6, all p > 0.08) at all sampling points throughout the trial.

To further test for possible differences in SSVEP amplitudes, we calculated the respective power by means of an FFT for a time window of 1500 ms starting at word onset. Results confirmed no amplitude differences between conditions (*Emotion*: F_1,17_ = 1.18, p = 0.29; *Lexicality*: F_1,17_ = 0.27, p = 0.61).

### Event related potentials

Figure 3 depicts the ERPs elicited by the change to a word or pseudoword and the respective difference maps. In the P2 time window (220–302 ms) an interaction of *RegionbyEmotion* (F_1,17_ = 7.0, p = 0.02, ƞ^2^ = 0.29) indicates that negative compared to neutral words elicited a larger positivity at frontal electrodes (*Emotion*: F_1,17_ = 5.7, p = 0.03, ƞ^2^ = 0.25; left: t_17_ = −2.3, p = 0.03; right : t_17_ = −1.8, p = 0.08) and more negative amplitudes over posterior sites (*Emotion:* F_1,17_ = 8.6, p < 0.01, ƞ^2^ = 0.34; left: t_17_ = 3.0, p < 0.01; right: t_17_ = 1.3, p > 0.2). The emotion effect was more pronounced over left hemisphere leads (*Region* × *Laterality* × *Emotion*: F_1,17_ = 3.4, p = 0.085, ƞ^2^ = 0.17).

Words had a larger deflection than pseudowords (*Region* × *Lexicality*: F_1,17_ = 7.9, p = 0.01, ƞ^2^ = 0.32), i.e. more positive amplitudes at frontal clusters (*Lexicality*: F_1,17_ = 6.0, p = 0.03, ƞ^2^ = 0.26, left: t_17_ = 2.2, p = 0.04, right: t_17_ = 1.9, p = 0.08) and more negative deflections at posterior clusters (*Lexicality*: F_1,17_ = 10.0, p < 0.01, ƞ^2^ = 0.37, left: t_17_ = −3.0, p < 0.01, right: t_17_ = −1.9, p = 0.08).

In the N400 time window (360–442 ms) an interaction of *Region* × *Emotion (*F_1,17_ = 10.7, p < 0.01, ƞ^2^ = 0.39) indicates that at posterior sites negative words led to more negative amplitudes than neutral words (*Emotion:* F_1,17_ = 11.8, p < 0.01, ƞ^2^ = 0.41, left: t_17_ = 2.6, p = 0.02, right: t_17_ = 2.8, p = 0.01), but more positive amplitudes at frontal electrodes (F_1,17_ = 9.4, p < 0.01, ƞ^2^ = 0.36, left: t_17_ = −2.9, p < 0.01, right: t_17_ = −2.3, p = 0.03). *Lexicality* of letter strings influenced the ERP amplitude as a function of *Region* (interaction: F_1,17_ = 13.6, p < 0.01, ƞ^2^ = 0.45) and *Laterality* (interaction: F_1,17_ = 7.9, p = 0.01, ƞ^2^ = 0.32). At frontal electrodes pseudowords led to more negative amplitudes (F_1,17_ = 11.2, p < 0.01, ƞ^2^ = 0.40), especially over the left hemisphere (*Laterality* × *Lexicality*: F_1,17_ = 8.6, p < 0.01, ƞ^2^ = 0.34, left: t_17_ = 3.7, p < 0.01, right: t_17_ = 1.9, p = 0.08). At posterior sites pseudowords led to more positive amplitudes compared to words (F_1,17_ = 15.7, p = 0.001, ƞ^2^ = 0.48). This effect was stronger at the right posterior cluster (*Laterality* × *Lexicality*: F_1,17_ = 3.4, p = 0.08, ƞ^2^ = 0.17, right: t_17_ = −4.7, p < 0.01; left: t_17_ = −1.9, p = 0.07).

In the LPC time window (480–645 ms) no significant effects including the factor *Emotion* were found (all |F| < 1, all p > 0.5). There was a main effect of *Lexicality* (F_1,17_ = 8.0, p = 0.01, ƞ^2^ = 0.32) and a significant interaction of *Region* × *Lexicality (*F_1,17_ = 3.7, p = 0.01, ƞ^2^ = 0.32). Meaningful words compared to pseudowords led to more negative amplitudes at frontal electrodes (F_1,17_ = 9.7, p < 0.01, ƞ^2^ = 0.36) and to more positive deflections at posterior sites (F_1,17_ = 5.5, p < 0.05, ƞ^2^ = 0.25). The effect was strongest at right anterior (t_17_ = −3.9, p < 0.01, left: t_17_ = −1.7, p = 0.1) and left posterior (t_17_ = 3.0, p < 0.01, right: t_17_ = 1.1, p = 0.3) electrodes.

## Discussion

We presented emotionally negative, neutral, and pseudowords in an LDT. Given the delayed response after word presentation, in contrast to previous studies [9, 33] behavioral results provided no informative effects but ensured that participants were engaged with the stimuli and in the task. Stimuli were presented at a flicker rate of 12.14 Hz to investigate whether emotional words facilitate neural activity at an early stage of stimulus processing, i.e. in the early visual cortex. If true, we expected greater 12.14 Hz SSVEP amplitudes elicited by emotional compared to neutral words. Emotion effects linked to later processing stages were expected to be refleced in an ERP modulation in the P2, N400 and LPC time windows.

Electrophysiological data provided clear evidence: SSVEP amplitude time courses as well as amplitudes integrated over 1.5 s of word presentation were identical for the three experimental conditions. Thus, the current results align with a previous report by Koban et al. [28], who reported no SSVEP amplitude differences between neutral and negative words during a presentation period of about 8 s as well as no differences in the time course analysis of SSVEP amplitudes between neutral and emotional words. However, in their study positive compared to neutral nouns elicited enhanced SSVEP amplitudes. Given the use of only negative nouns and the compareably short trial duration in the current experiment, our results cannot elaborate on this finding. Because the first second of stimulus presentation was not analyzed by Koban et al. [28], we suggest, in line with the authors, that, instead of ‘motivated attention’ [2], slow processes of evaluation, emotion regulation, or mental imagery may underlie this late effect. Together with the “null finding” when comparing words and pseudowords, the present results suggest that neither semantic nor affective word content captured visual attention as has been reported for pictoral stimuli [4, 23, 26, 27].

In contrast, in the AB experiment by Keil et al. [29] SSVEP amplitudes were modulated for emotionally negative words presented at a ‘lag’ usually denoting the AB. One big difference between this study and the current one is that words were presented in an RSVP with each cycle presenting a different word, while in the Koban et al. [28] and the present study one word was presented per trial. Due to the short stimulus presentation (50 ms) and the requirement to actively read the words more attentional resources may have been deployed by the RSVP. On the other hand, statistically significant SSVEP amplitude modulations were only observed for second targets (T2) during the AB, and, as discussed by the authors, the lag resulted in a superimposition of the second peak that was elicited by T1 and the first peak for T2 (i.e. the respective emotional/neutral word). Perhaps the high attentional demands within a short time window of 230 ms (onset T1 to onset T2) contributed to this finding. An alternative explanation for the emotion effect could be the P2 amplitude elicited by T2, given that the steep peak of the P2 may contribute to the 8.6 Hz SSVEP signal. In the present study we found an enhanced P2 amplitude for negative compared to neutral words (see “Discussion” below) and latency and topography of the effect seem to roughly overlap with the SSVEP effect reported by Keil et al. [29]. Therefore, such an additive effect seems possible. Against this alternative explanation speaks the fact that SSVEP amplitudes were only different at ‘lag 2’ and additive effects of SSVEP and P2 should occur for T1 stimuli and T2 stimuli at later lags as well. However, ERPs were not reported for the RSVP experiment. It remains an open question whether attentional demands (e.g. with short presentation times or degraded stimuli) may mediate an effect of emotional words on early visual cortex.

The absence of a significant SSVEP modulation by emotional words is in accordance with our previous study [21], where words were presented as irrelevant background stimuli and the SSVEP was elicited by a demanding visual task overlapping these words. Given that words were task-relevant in the present study and the SSVEP was elicited by the word form rather than competing stimuli, the present results strengthen the notion that affective words may not capture additional early visual processing resources.

Relating the present SSVEP result to prior work concerning emotion effects on early visual areas, we first turn to P1 effects. Only a couple of studies report P1 modulations by emotional words [6, 7]. Some of these results are related to additional variables such as gender [34] or conditioning [35]. Recent research focussed on the contributions of valence and arousal and yielded mixed results, i.e. P1 effects of highly arousing words [10] in contrast to effects of only negative [9] or only positive words [36]. Whilst the influence of arousal and valence or differential motivational systems for positive and negative affect remain a matter of debate [7], it is assumed that effects in early visual areas are a consequence of rapid lexical access [9, 10]. It may therefore be useful to study the influence of high- versus low-frequency emotional words on the SSVEP. Interestingly, Hofmann and colleagues [10] corroborate this assumption with source localisation of the P1 effect in the fusiform area, along with midtemporal regions. Keuper et al. [37] also located the source of an effect of emotional words on the P1 in midtemporal structures, close to one proposed major source of SSVEP responses (V5, [22]). Ortigue et al. [38] found bilateral occipital sources for enhanced activity towards negative words in a hemifield paradigm. There may be overlap in the cortical sources of P1 and SSVEP signals. Still, given that early (<200 ms) visual ERP effects are not a robust finding for emotional words [6, 7], the absence of an SSVEP effect in the present study seems not surprising. The current findings, however, provide complementary evidence to prior ERP research, because the two measures reflect at least partially distinct mechanisms [39] and the SSVEP as a continuous measure, in contrast to early ERP components as well as imaging studies, provides temporal information about the allocation of attention.

In line with the mixed findings in the P1 amplitude modulations, some imaging studies reported effects in occipital cortex [40, 43], whereas others found effects of emotional word content in temporal and frontal cortices, but not in inferior occipital areas [41, 42]. Schlochtermeier et al. [43] provide interesting evidence, that visual complexity may account for differential emotion effects by pictoral compared to word stimuli in perceptual processing regions. Given mixed results in previous research, the present findings support the idea that written emotional words are not necessarily facilitated at an early visual processing level. Open questions about the role of attentional demands, visual complexity or word frequency for this assumption could also be adressed using the SSVEP in future studies on emotional word processing.

Turning to our ERP results, we found typical ERP modulations [6, 9, 33] from 200 ms onwards even though the emotional word content was task irrelevant. The P2 seems to indicate early lexical and semantic access [6, 44]. In line with this assumption, the frontal P2 in the present study was enhanced for words compared to pseudowords. During this time window emotional words have been found to elicit an enhanced frontal positivity [17, 18, 45] indicating facilitated lexical access for emotional words. Other studies report a left-lateralized early posterior negativity (EPN, [16, 46]) interpreted as reflecting attentional capture by emotional stimuli [47]. We replicated both findings here as well as in a previous study [21].

In spite, or because, of their different scalp distribution and polarity, P2 and EPN overlap in latency and their sensitivity to emotional word content and can be observed in parallel. This raises the question whether they arise from the same underlying neural process. In search of a more integrative view we also relate the pattern and latency of the present emotion effect to previous reports on the recognition potential (RP), a frontal positivity [48] and/or posterior negativity [49] peaking at about 250 ms, which is sensitive to visual features as well as the semantic content of word forms and pictures. There is some debate about whether the RP reflects visual or semantic processing [14]. It may originate in the visual word form area and is sensitive to visual as well as semantic manipulations. The RP is therefore best described as a process of matching complex visual information to stored representations by specialized object recognition units (for words, faces, or pictures). Given that perceptual features, i.e. the visual complexity of word forms, were rather constant in our study, effects in the P2 time range indicate that arousing words enhanced early stages of lexico-semantic access rather than initial perceptual analysis.

Modulations of the P2 by emotionally arousing words have also been reviewed by Kotz and Paulmann [19]. Whilst the authors discussed enhancement of the P2 as reflecting selective attention towards arousing words, they also emphasize a distinction between lexico-semantic integration reflected in the P2 time range from sensory processing stages (as the N100 component towards auditory stimuli or the SSVEP elicited by word forms in the current experiment). Therefore we interpret the present emotion effect in the P2 time range as indicative of enhanced lexico-semantic access.

The N400 is associated with higher-order lexico-semantic processing and semantic integration as it responds sensitively to expectations (e.g. word status: how probable a certain word is to conclude a sentence) or semantic anomalies as in the case of pseudowords, reviewed e.g. in [20]. Accordingly, an enhanced frontal N400 to pseudowords was replicated here indicating enhanced processing effort with meaningless word forms. As the present experiment did not systematically establish a semantic context it seems not surprising that the component is more transient and less pronounced than previously reported N400 effects in sentence contexts [20]. Negative words elicited a smaller frontal negative response than neutral words. Paralleling previous findings of a decreased N400 amplitude to negative nouns [17, 50] the present result supports previous evidence that affective word content seems to facilitate late stages of refined and integrative semantic processing. However, Herbert et al. found a decreased N400 amplitude only for positive compared to negative adjectives during silent reading [16]. Thus, the impact of word type, task, and emotional valence on the N400 component remain to be debated.

The LPC was unaffected by emotional word content, instead it responded to *Lexicality*. A larger late centroparietal positivity to words compared to pseudowords is thought to reflect ongoing evaluative processing of semantically rich stimuli [44]. Late emotion effects in the ERP have repeatedly been found to depend on task demands. Fischler et al. reported that emotional words enhanced the LPC in a semantic categorisation task but not in an LDT [51]. Schacht and Sommer [52] found an emotion effect on the LPC with a semantic task as well as with an LDT, but not with a shallow visual task. However, in their LDT only positive words differed from neutral words in LPC amplitude, no significant effect was reported for negative stimuli [52]. Similar results were reported by Herbert et al. [16] in a silent reading task: only positive words differed from neutral and negative words in terms of LPC amplitude. In contrast, Hinojosa and colleagues [53] found larger LPC amplitudes for negative compared to neutral words in an LDT. As concluded in a recent review by Citron [7] mixed results concerning emotion effects on the LPC may be explained by differences in tasks and stimulus features (nouns/adjectives/verbs, high- or low-frequency words). In contrast to emotion effects in the P2/EPN time range, the LPC seems to reflect more cotrolled processing processing of emotional valence [7].

## Conclusions

In sum, the present ERP results suggest that emotional word content enhanced lexico-semantic access (P2 time range) and facilitated subsequent semantic processing (N400 time range), whereas the LPC appeared indicative of task-related evaluative processing. In contrast, the SSVEP as a measure of activity in early visual cortex was insensitive to affective or lexical word content. We conclude that the present results provide further evidence that emotional word content may modulate processing of word forms subsequent to lexical access without, unlike emotional pictures or faces [4, 23, 26, 27], enhancement of early visual stimulus processing. SSVEP amplitudes seem to represent a useful measure to further disentangle the prerequisites of emotion effects on early visual cortex activity.
